# Evaluation of Genotoxic Effect and Antigenotoxic Potential against DNA Damage of the Aqueous and Ethanolic Leaf Extracts of *Annona muricata* Using an In Vivo Erythrocyte Rodent Micronucleus Assay

**DOI:** 10.1155/2022/9554011

**Published:** 2022-12-16

**Authors:** Susana Vanessa Sánchez-De-La-Rosa, Blanca Patricia Lazalde-Ramos, Gabriela Morales-Velazquez, Guillermo Moisés Zúñiga-González, Belinda Claudia Gómez-Meda, Saulo Oswaldo Sánchez-Rivera, Yveth Marlene Ortiz-García, Celia Guerrero-Velazquez, Ana Lourdes Zamora-Perez

**Affiliations:** ^1^Instituto de Investigación en Odontología, Centro Universitario de Ciencias de la Salud, Universidad de Guadalajara, Guadalajara 44340, Mexico; ^2^Maestría en Ciencias y Tecnología Química, Unidad Académica de Ciencias Químicas, Universidad Autónoma de Zacatecas, Zacatecas 98000, Mexico; ^3^Laboratorio de Mutagenesis, Centro de Investigación Biomédica de Occidente, Instituto Mexicano del Seguro Social, Guadalajara 44340, Mexico; ^4^Instituto de Genética Humana “Enrique Corona Rivera” Departamento de Biología Molecular y Genómica, Centro Universitario de Ciencias de la Salud, Universidad de Guadalajara, Guadalajara 44340, Mexico

## Abstract

*Annona muricata* have been extensively used in traditional medicine to treat multiple diseases, including cancers. This study evaluated the genotoxic potential and antigenotoxic activities of *A. muricata* aqueous and ethanolic leaf extracts by employing an in vivo erythrocyte rodent micronucleus assay. Different doses (187.5, 375, and 750 mg/kg) of both extracts were administered orally for 5 days alone and combined with cyclophosphamide (CP, 60 mg/kg) to BALB/c mice. Also, it was administered orally to Wistar rats for 5 days through the final stage of gestation. No genotoxic or cytotoxic effects were observed in the two adult rodent models when *A. muricata* was administered orally nor in newborn rats transplacentally exposed to the extracts. Moreover, *A. muricata* aqueous and ethanolic leaf extracts demonstrated a protective effect against CP-induced DNA damage. Due to its lack of genotoxic effect and its capacity to decrease DNA damage, *A. muricata* is likely to open an interest field regarding its potential safe use in clinical applications.

## 1. Introduction

Medicinal plants are used for treating diseases and as a source of bioactive compounds for developing drugs and dietary supplements. In recent decades, the consumption of these plants as well as supplements containing the whole plant or plant extracts has increased in the general population as an alternative or adjunctive treatment to reduce the use of synthetic drugs [1].

*Annona muricata*, also known as guanabana, graviola, or soursop, is a native plant of Central and South America [2]. This plant is from the genus Annona, and the Annonaceae family; *A. muricata* is the most widely known and studied [2]. Extracts from different parts of the plant such as the bark, root, pulp, seeds, and leaves have been used for medicinal purposes [2]. The decoction of the leaves is the most used preparation [2]. Traditionally, it has been used to treat diarrhea, malaria, colds, hypertension, and cancer. Also, *A. muricata* extracts have been the subject of several studies regarding their pharmacological properties. For example, ethanolic and methanolic extracts showed antibacterial activities in vitro [3], including antiprotozoal activity [3], and in *in vivo* studies, the methanolic and aqueous extracts showed hypoglycemic effects in a murine model [4]. Moreover, *A. muricata* has been studied for its antioxidant activities since these effects have been shown to be mediated by phenols, flavonoid carotenoids, and vitamins it contains [5]; thus, most of the pharmacological studies focus on its cytotoxic activity. The ethyl acetate and ethanolic extracts of *A. muricata* leaves have shown cytotoxicity in different tumor lines in both *in vitro* and *in vivo* studies [6]. Regarding its genotoxic and cytotoxic activities, Mutakin et al. found no genotoxic or cytotoxic activity of the hydroalcoholic extract of *A. muricata* leaves in mouse bone marrow using the micronucleus assay [7].

*A. muricata* contains several compounds that are known for their antigenotoxic and free radical scavenging activities [8, 9], as well as the extract of the leaves is known for its cytotoxic and anticancer properties [10].

Most extracts and products derived from medicinal plants have not undergone toxicological studies to which pharmacological compounds are subjected, because it is traditionally used, and thus, a nontoxic effect is assumed [2, 7].

Nonetheless, medicinal plants' consumption of medicinal plants for the treatment of many diseases could cause important genotoxic, cytotoxic, and teratogenic effects [11]. Therefore, the potential genotoxic, cytotoxic, and antigenotoxic activities of *A. muricata* leaf extracts need to be assessed due to their typical and increasing consumption as a medicinal agent.

Citogenotoxic and antigenotoxic assays have been used to detect the effects of compounds and environmental factors on the genetic material to identify potential anticancerigens and antimutagens, including the development of new drugs to be used in the prevention and treatment of diseases [8]. Different bioassays were used to evaluate DNA damage *in vivo* with clear and accurate results, such as the micronuclei (MN) assay in rodent peripheral blood [9]. This test is included in the basic *in vivo* genotoxicity testing that evaluates the safety of compounds. MN are fragments or complete chromosomes that spontaneously or as a result of DNA damage are excluded from the nucleus. Therefore, MN indicates chromosomal breakage or loss [10].

On the other hand, various chemicals have the potential to induce congenital disabilities [12]. It should be noted that compounds that are classified as genotoxic may have teratogenic potential, and several mechanisms of teratogenesis could involve the induction of MN [9, 13].

**Figure 1:**
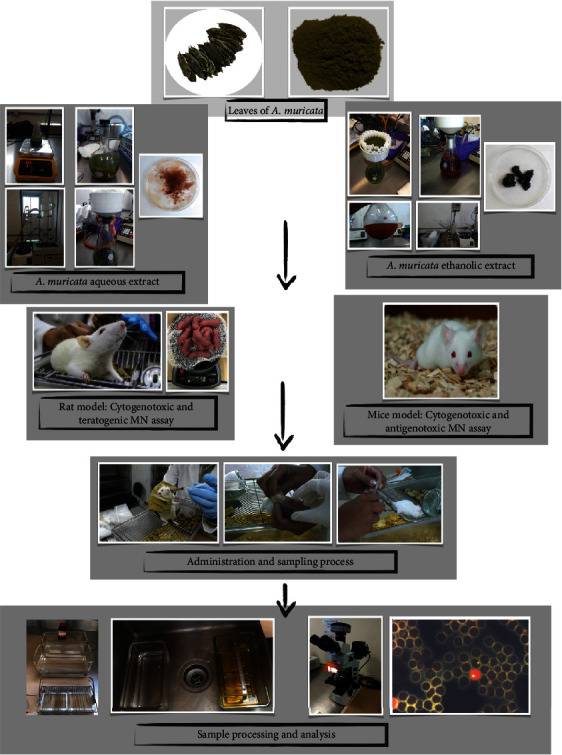
Overview of the study.

Considering the increased consumption of *A. muricata* in folk medicine and the lack of previous research regarding its cytotoxic and antigenotoxic properties, the present study focuses on the in vivo analysis of the potential genotoxic effects of *A. muricata* aqueous and ethanolic leaf extracts utilizing an erythrocyte rodent micronucleus assay as well as an initial analysis of its antigenotoxic activities against CP-induced DNA damage because this clastogenic and alkylating agent, during its metabolization, interacts with DNA and induces the formation of DNA adducts that cause oxidative DNA damage. The overview of this study is represented in Figure 1.

## 2. Materials and Methods

### 2.1. Plant Material

The leaves of *A. muricata* were obtained and authenticated by The Greens Shop Company (Miguel Aleman #128 Colonia Centro, Veracruz, Mexico).

### 2.2. Preparation of the Aqueous and Ethanolic Leaf Extract of *A. muricata*

The dried plant leaves were ground to a fine powder with a particle size of less than 0.5 mm. The aqueous extraction was carried out by the decoction method. The powder was dissolved in a ratio of 1 g per 10 mL of water, boiled for 5 min, and then filtered to eliminate the bagasse. Activated carbon was added to the resulting filtrate, and it was macerated for 48 h to remove pigments before filtering once again and the remaining material left was lyophilized.

The preparation of the ethanolic leaf extract of *A. muricata* was carried out by mechanical maceration in a proportion of 70 g of the plant in 300 mL of 70% ethanol for 48 h. The mixture was refluxed (62°C) for 2 h and filtered. Activated carbon was added to the resulting filtrate, and it was macerated for 48 h to remove pigments before filtering once again. Ethanol was removed using a rotary evaporator and subsequently lyophilized. The samples were stored at a refrigerated temperature of 4°C for further use.

### 2.3. Doses of Aqueous or Ethanolic Leaf Extracts of *A. muricata* Used in the In Vivo Assays

The doses of 187, 375, and 750 mg/kg of aqueous or ethanolic leaf extracts of *A. muricata* used in the animals testing were based on previous reports in mice and rats that focused on evaluating biological activity [14].

### 2.4. Animals

In this study, 2.5-month-old BALB/c male mice 20 g approx. (average weight 26.04 ± 1.78 g) and 3-month-old female Wistar rats 200 g approx. (average weight 183.90 ± 26.15 g) were used. Elimination criteria included female rats that did not mate. All animals were provided by the animal facilities of the *Centro de Investigación Biomédica de Occidente (Instituto Mexicano del Seguro Social)*, Guadalajara, Jalisco, Mexico.

Animals were housed in polycarbonate cages in windowless rooms, with automatic temperature control (22 ± 2°C) and lighting (lights on at 07:00 and off at 19:00 h) and maintenance of relative humidity (50 ± 10%). Animals received standard laboratory pellet food (Purina®, Mexico) and tap water ad libitum.

This study was approved (registration no. CII9/2016) by the Research Ethics Committee of the University of Guadalajara.

### 2.5. Study Groups and Micronuclei Induction in Mice

**Figure 2:**
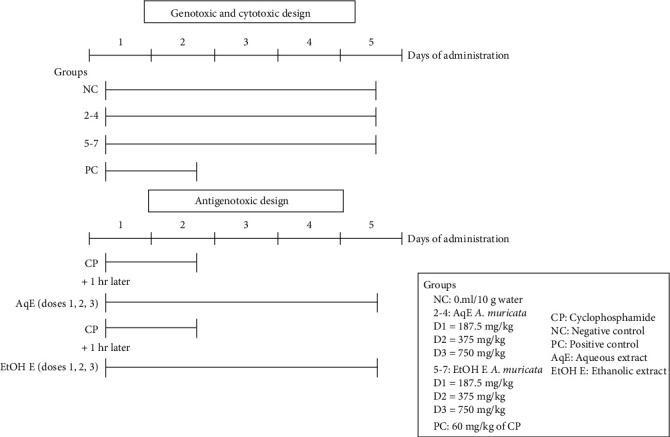
Experimental design to evaluate *in vivo* the genotoxicity, cytotoxicity, and antimutagenicity of *A. muricata* aqueous and ethanolic leaf extracts in mice peripheral blood. Genotoxic and cytotoxic evaluation. Groups: negative control sterile water for five days; 2-7: one of the following doses (187.5, 375, and 750 mg/kg) of *A. muricata* aqueous and ethanolic leaf extracts for 5 days; PC: positive control 60 mg/kg of CP divided into two doses of 30 mg/kg, once daily for two days. Antigenotoxic evaluation. Groups: mice given 1 h prior to CP administration (60 mg/kg of CP divided into two doses of 30 mg/kg) once daily for two days one of the following doses (187.5, 375, and 750 mg/kg) of aqueous and ethanolic leaf extracts of *A. muricata* once daily for 5 days.

In total, 70 male mice were used, and through an experimental design to evaluate the genotoxic, cytotoxic, and antigenotoxic effects of aqueous or ethanolic leaf extracts of *A. muricata*, 14 groups were formed randomly using simple randomization, 5 mice/group/per cage in accordance with MN protocol for *in vivo* rodent assay [15], as follows (Figure 2):

- Group 1: negative control (NC), mice were given sterile water once daily for 5 days
- Groups 2–7: genotoxic and cytotoxic evaluation of aqueous or ethanolic leaf extracts of *A. muricata*, mice were given one of the following doses (187.5, 375, and 750 mg/kg) of aqueous or ethanolic leaf extracts of *A. muricata* once daily for 5 days
- Groups 8–13: antigenotoxic evaluation of aqueous or ethanolic leaf extract of *A. muricata*, mice were given 1 h prior to the administration of 60 mg/kg of CP divided into two doses of 30 mg/kg of cyclophosphamide (CP; CAS 6055-19-2 Sigma-Aldrich, St. Louis, MO, USA) once daily for two days one of the doses of aqueous or ethanolic leaf extracts of *A. muricata* once daily for 5 days
- Group 14: positive control (PC), mice were given 60 mg/kg of CP divided into two doses of 30 mg/kg, once daily for two days

The administration volume of all doses was 0.1 mL/10 g of weight, and these were administered orally with an esophageal cannula. All procedures were performed in accordance with current guidelines, and the investigators in charge of the administration were not aware of group allocation [15].

### 2.6. Sampled Preparation and Micronuclei Analysis in Mice

The evaluation of genotoxic, cytotoxic, and antigenotoxic effects was determined by the MN assay. Animals were sampled every 24 h for 6 days [15, 16] after administering of the corresponding dose. A small excoriation was made to take blood samples from the tip of the tail, and two smears were obtained and placed on precoded slides. The investigator in charge of sampling was the only one aware of the group assignment. Samples were dried and fixed in absolute ethanol (Brands Golden Bell, Mexico, and J. T Baker Mexico) for 10 min and stained with acridine orange (CAS 10127-02-3, Sigma-Aldrich, St. Louis, MO, USA) for analysis [17]. All samples were scored manually and without previous knowledge of group assignment, using an Olympus microscope CX31 equipped with epifluorescence and an oil-immersion objective (100x).

The parameters analyzed included the number of micronucleated polychromatic erythrocytes (MNPCEs) per 1000 polychromatic erythrocytes (PCEs) to assess recent damage produced 24 h before sampling, the number of MNEs per 10000 total erythrocytes (TEs) to evaluate the damage accumulated during the exposure time, and the number of PCEs in 1000 TEs was also determined as a control system since a reduction in the number of PCE number reveals bone marrow toxicity [15].

**Figure 3:**
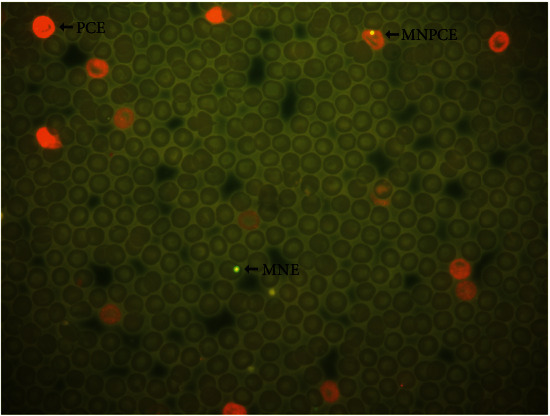
Fluorescent microphotograph of MNPCEs, MNEs, and PCEs from a *A. muricata* leaf extract-treated rodents. Arrows indicates MN in an immature (polychromatic) erythrocyte (PCE) and in a mature (normochromatic) erythrocyte (NCE). PCE are stained in orange, MN in yellow, and NCE in dark green. Erythrocytes (PCE or NCE) without MN are considered normal cells (stain acridine orange; 100x).

The antigenotoxic effect of *A. muricata* aqueous or ethanolic leaf extract against the damages caused by the CP was evaluated counting 1000 PCEs per animal and analyzing the MNPCE frequency and 10000 TEs per animal and analyzing MNE frequency [15]. With acridine orange staining, mature erythrocytes were identified by their green color, immature erythrocytes (PCEs) by their orange–red color, and MN by their yellow color (Figure 3).

### 2.7. Mating of Rats

**Figure 4:**
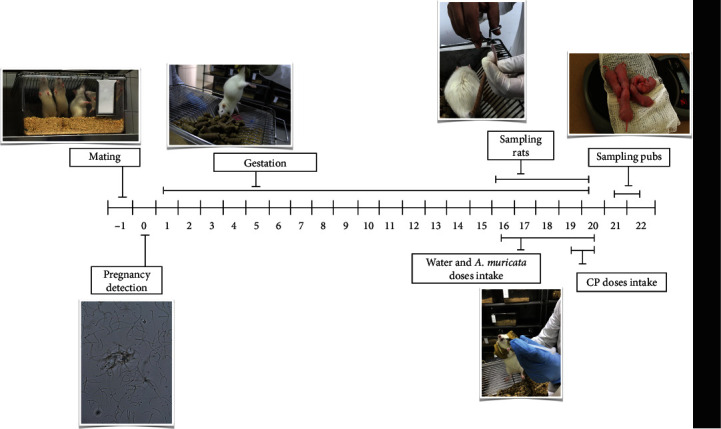
Schematic representation of transplacental MN assay in rats.

Fifty female rats were mated with males in cages containing three females and one male. Each female rat was flushed daily with a vaginal wash of 0.1 mL of sterile water using an adjustable volume pipette. The contents were smeared onto clean slides, which were analyzed without stain using a light microscope. The day of the initial identification of sperm was established as the first day of the pregnancy, after which female rats were housed in individual cages, and a dose administration schedule was assigned for each rat [18]. Ten rats were eliminated because the presence of sperm was not identified. (Figure 4).

### 2.8. Study Groups and Micronuclei Induction in Rats

Rats were divided by simple randomization, distributed into five groups (five rats per group; each rat was housed in an individual cage, following MN protocol for *in vivo* rodent assay [15] as follows:

- Group 1: NC, rats received sterile water once daily for 5 days
- Group 2–6: rats given one of the following doses (187.5, 375, and 750 mg/kg) of aqueous or ethanolic leaf extracts of *A. muricata* once daily for 5 days
- Group 7: PC, 60 mg/kg of CP

The doses were administered orally with an esophageal cannula to each rat throughout the final stage of the gestation period (days 16 to 20 of the gestation process) [18]. The dose of CP was administered in two doses of 30 mg/kg in days 19 to 20 of the gestation process. All doses were administered at a volume of 0.1 mL/10 g of weight. Furthermore, the investigators in charge of the administration were unaware of group allocation.

### 2.9. Sample Preparation and Micronuclei Analysis in Rats

Blood samples were taken from pregnant rats by a small cut from the tip of the tail. Samples were collected before the first administration (basal sample) and every 24 h for 6 days, from gestational day 16 to day 21 until delivery [18] at 0, 24, 48, 72, 96, and 120 h. At birth (from day 21 and 22), six pups per rat were selected randomly and weighed, and a drop of blood was obtained from each pup from the tip of the tail to make two smears on precoded slides. Samples were processed as described for mice.

For the pup's blood sample analysis, the number of MNEs in 10000 TEs and MNPCEs in 1000 PCEs were counted, and the proportion of PCEs in 1000 TEs in all samples was determined. In the case of adult rats, MNPCEs in 3000 PCEs were counted, and the proportion of PCEs in 1000 TEs was determined [19] (Figure 4). Adult rats have very few MNEs in the peripheral blood; therefore, the MN assay must be performed using the bone marrow and/or by counting MNPCEs in the peripheral blood.

### 2.10. Statistical Analysis

Data are expressed as the mean ± standard deviation of MNE, MNPCE, and PCE frequencies. The Kolmogorov–Smirnov test was used to establish that the data were normally distributed. In the case of mice and pregnant rats, intragroup comparisons were performed between each treatment group and their respective basal value (0 h) using repeated measures ANOVA, followed by a Bonferroni test to correct the significance values of the multiple post hoc pairwise comparisons.

For newborn rats, the litter was used as the experimental unit (*n* = 6/group), and pups were the unit of observation and analysis. One-way ANOVA, followed by the Dunnett *t*-test for multiple post hoc pairwise comparisons versus the appropriate control, was used to correct the intergroup analysis significance values. A *p* value of less than 0.05 was considered significant. Data analysis was performed using IBM SPSS (V25) statistics program for Windows.

The antigenotoxic effect is expressed as a percentage of damage reduction (%DR): the percentage that the genotoxic agent-induced damage was reduced by *A. muricata* according to Waters et al. [20] and Fedato and Maistro [21] using the following formula:

(1) $$\begin{array}{c} \%\text{DR}=\left(\frac{A-B}{A-C}\right)\times 100, \end{array}$$

where *A* corresponds to the MNPCE or MNE mean in the group treated with CP (PC group), *B* corresponds to the MNPCE or MNE mean in the antigenotoxic treatment (*A. muricata* extract plus CP), and *C* corresponds to the MNPCE or MNE mean in the NC group.

### 2.11. Ethical Considerations

All experiments were performed following the guidelines for the use and care of research animals specified in the regulations and national norms (Official Mexican Standard NOM-062-ZOO-1999), including the specifications and techniques for the production, care, and use of institutional laboratory animals and the International Institutes of Health for the humane treatment of research animals. Furthermore, animals were sacrificed according to the NOM-033-SAG/ZOO-2014 and their remains were treated as indicated by the NOM-087-ECOL-SSA1-2002 for Environmental Protection-Environmental Health-Infectious Biological Hazardous Waste-Classification and Handling Specifications. Management of animal use followed the principles and guidelines approved by the Guide for the Care and Use of Laboratory Animals, while euthanasia followed the CONCEA Euthanasia Practice Guidelines. The euthanasia at the end of the experiments was performed by anesthesia with an intraperitoneal injection of ketamine (100 mg/kg) which was followed by an intracardiac injection of potassium chloride (0.05 mL).

## 3. Results

### 3.1. Genotoxic, Cytotoxic and Antigenotoxic Effects of Aqueous or Ethanolic Leaf Extract of *A. muricata* in Mice

Tables 1–3 summarize the results of the genotoxic and cytotoxic effects of the three doses of the aqueous and ethanolic leaf extracts of *A. muricata* and controls in mouse peripheral blood.

Genotoxicity was evaluated by counting the number of MNPCEs and MNEs in the peripheral blood of mice. There were significant increases in MNPCEs (*p* < 0.05; from 24 until 120 h) and MNE frequencies (*p* < 0.05; from 48 h until 96 h) in the PC group compared to baseline values (Tables 1 and 2), confirming the sensitivity of the test. In contrast, in the NC and experimental groups, the three tested doses of the aqueous and ethanolic leaf extracts of *A. muricata* did not induce significant changes in the number of MNPCEs and MNEs in any treatment period (Tables 1 and 2).

Cytotoxicity was monitored by determining proportion of PCEs in the mouse peripheral blood (Table 3). In the NC and experimental groups, 187.5, 375, or 750 mg/kg dose tested of aqueous and ethanolic leaf extracts did not present significant changes in the proportion of PCEs, concerning their baseline values. In contrast, the PC group showed significant decreases (*p* < 0.05) in the proportion of PCEs at 72, 96, and 120 h (Table 3).

The antigenotoxic effect and the %DR of *A. muricata* aqueous and ethanolic leaf extracts against CP-induced damage in the number of MNPCEs and MNEs are shown in Tables 4 and 5.

In the present work, all groups treated with one of the three doses of the aqueous or ethanolic leaf extracts of *A. muricata* and CP significantly reduced the frequency of MNPCEs and MNEs compared to the PC group, showing that the aqueous and ethanolic extract of *A. muricata* present antigenotoxic activity under these experimental conditions (Tables 4 and 5).

Examining the antigenotoxic and %DR results, the evaluated doses of aqueous leaf extracts of *A. muricata* decreased MNPCEs significantly (*p* < 0.05) in mice treated with the lowest dose at 48 h to 72 h and the %DR were 61.22 and 67.50, respectively; the middle dose at 48 h (%DR 42.86), 72 h (%DR 62.50), and 96 h (%DR 55.81); and the highest dose at 96 to 120 h by 55.81%DR and 11.11%DR, respectively (Tables 4). Moreover, the evaluated doses of ethanolic leaf extracts of *A. muricata* decreased MNPCEs significantly (*p* < 0.05) with the lowest dose at 72 h to 120 h and the %DR were 68.75, 72.09, and 61.11%, respectively; the middle dose at 48 h (%DR 46.94), 72 h (%DR 67.50), and 96 h (%DR 65.12); and the highest dose at 72 to 120 h by 70.00%DR, 67.44%DR, and 33.33%DR, respectively (Table 4).

Additionally, MNE frequency decreased significantly (*p* < 0.05) with the different doses of aqueous leaf extracts of *A. muricata* at 72 to 120 h. The %DR with the lowest dose were 54.82% (72 h), 84.21% (96 h), and 46.38% (120 h), with the middle dose 86.80%, 81.95%, and 62.32%, respectively, and in with the highest dose 68.02%, 69.17%, and 40.58%, respectively (Table 5). Likewise, the doses of ethanolic leaf extract of *A. muricata* diminished the number MNE significantly (*p* < 0.05) with the lowest dose at 96 h to 120 h and the %DR were 65.41 and 60.87%, respectively; the middle dose at 48 h to 96 h with a %DR of 70.89, 65.48, and 57.89%, respectively; and the highest dose at 24, 72, and 96 h by 58.49%DR, 57.36%, and 43.61% correspondingly (Table 5).

### 3.2. Genotoxic and Cytotoxic Effects of Aqueous or Ethanolic Leaf Extracts of *A. muricata* in Pregnant Rats

In pregnant rats, the mean weight of pregnant rats before treatment (at day 16 of pregnancy) was 235.37 ± 19.73 g, the average number of offspring per litter was 11.23 ± 0.43 pups, and the average weight of pups at birth was 6.22 ± 0.02 g, with no significant differences among groups.

Tables 6 and 7 summarize the results of the genotoxic and cytotoxic effects of the three doses of the aqueous and ethanolic leaf extracts of *A. muricata* and controls in pregnant rats.

Genotoxicity was evaluated by counting the number of MNPCEs in the peripheral blood of pregnant rats. In the intragroup comparison of MNPCEs, no significant changes were detected in either the NC group or the six experimental groups exposed to the aqueous or ethanolic leaf extracts of *A. muricata* in any treatment (Table 6). However, the number of MNPCEs in the PC group showed significant differences (*p* = 0.001) from 96 h until 120 h (Table 6).

Cytotoxicity was monitored by determining the proportion of PCEs in the peripheral blood of pregnant rats (Table 7). When PCE values were analyzed in the intragroup comparisons, a significant decrease (*p* < 0.05) at 96 h and 120 h was only observed in the PC group (Table 7).

### 3.3. Genotoxic and Cytotoxic Effects in Newborn Rats after Transplacental Exposure to *A. muricata* Aqueous and Ethanolic Leaf Extracts

In pups of rats, genotoxic and cytotoxic results of the intergroup comparisons of MNPCEs, MNEs, and PCEs, from the different study groups are shown in Table 8.

In the evaluation of transplacentally exposed pup samples to *A. muricata* aqueous and ethanolic leaf extracts, we observed a significant (*p* = 0.001) increase in MNPCE and MNE frequencies and a significant (*p* = 0.001) decrease in PCE between the PC group (*p* = 0.001) when compared with the NC group. However, no significant increases were observed in the number of MNPCE nor MNE number and no significant reductions in PCE frequency, when comparing the NC group with the groups treated with the different doses of *A. muricata* aqueous and ethanolic leaf extracts (Table 8).

## 4. Discussion

Our research demonstrated that *A. muricata* aqueous and ethanolic leaf extracts do not exert citogenotoxicity effect in adult mice, pregnant rats, or in the offspring born to the pregnant rats. Also, the analysis of the effect of the extracts on genotoxicity induced by CP shown that the three different doses of the aqueous and ethanolic leaf extracts of A. muricata were able to reduce CP-induced DNA damage under in vivo conditions.

The results obtained in this research are relevant since using medicinal plants to treat many diseases is a common practice in primary healthcare due to their low cost and easy availability. Also, consuming natural products may avoid the harmful effects of toxic environmental compounds and prevent multiple human diseases [22]. Nevertheless, traditionally, natural remedies are considered safe for treatment because of their natural origin, and consequently, they are widely used for self-medication [23]. In this context, increasing attention is given to the possible genotoxicity, cytotoxicity, and antigenotoxicity of natural products since toxicological evaluations are crucial to determine the range of doses and obtaining safety information for their use as an alternative medicine [22, 23].

*A. muricata* has been widely used as a traditional medicine for many disorders such as skin and respiratory disease, fever, bacterial infections, diabetes, hypertension, and cancer [1, 2]. Different parts of *A. muricata* have different activities, and in particular, the leaves are traditionally used as ethnomedicine to treat cystitis, headaches, insomnia, diabetes, hypertension, and cancer [2]. More than 200 chemicals have been identified and isolated from this plant. *In vitro* studies have characterized *A. muricata* as an antioxidant, anti-inflammatory, and cytotoxic substance in tumor cells. *In vivo* studies of the crude extract of this plant were shown to present antistress, antitumoral, hepatoprotective, and hypoglycemic activities [2]. Thus, this study evaluated not only its genotoxic potential but also a possible protective activity against DNA damage induced by a chemical mutagen as CP.

In the present work, three different doses of *A. muricate* aqueous and ethanolic leaf extracts (187.5, 375, and 750 mg/kg) were evaluated for and exposure period of 5 days to determine the genotoxic, cytotoxic, and antigenotoxic effects of this plant in rodent peripheral blood utilizing *in vivo* MN assay. MN are well-known markers of DNA damage, and this test is widely used in *in vivo* experiments to assess the genotoxic potential of any compound [24, 25].

According to our results, the aqueous and ethanolic leaf extracts of *A. muricata* did not increase MNE or MNPCE (chromosomal break or damage in the spindle apparatus) frequencies or decrease the proportion of PCEs in adult mice, pregnant rats, or in the offspring born to the pregnant rats at all doses and treatment periods, suggesting that *A. muricata* leaf extracts has no genotoxic (anegeunic and/or clastogenic) nor cytotoxic effects in any of the doses tested in the rodent peripheral blood.

Our results are in accordance with those reported by Acésio et al., where the antioxidant, cytotoxic, and genotoxic potentials of the hydroalcoholic extract of *A. muricata* leaves were evaluated *in vitro* and *in vivo*. Regarding genotoxicity, the authors report that after treatment with four concentrations (0.125, 0.25, 0.5, and 1 *μ*g/mL) of the hydroalcoholic leaf extracts of this plant, no significant differences in MN frequency and negative control groups were observed in V79 cell cultures. Also, *in vivo*, mouse bone marrow cells have found no significant differences in the MNPCE frequency between animals treated with different *A. muricata* leaf extract doses compared to the negative control [26]. However, contrary to our results, Acésio et al. reported significant cytotoxicity of *A. muricata* leaf extracts by the clonogenic assay; they reported that concentrations of ≥8 *μ*g/mL of *A. muricata* leaf extracts significantly reduced V79 cell viability compared to the negative control [26]. These differences may be since cytotoxicity was evaluated in vivo in the present work, thus showing fewer PCEs. Also, the lack of cytotoxic effect could be explained by acetogenins, attributed to which cytotoxic effects have been reported to have low bioavailability [27]. The proportion of PCEs in the peripheral blood can be altered by receiving a cytotoxic compound, MN formation, or an increased DNA damage, indicating cell death and myelosuppression [25]. The absence of cytotoxicity observed in the present work after five days of exposure of the selected doses of *A. muricata* leaf extracts is important; however, it does not confirm the safety of the tested substance [28]. In this study, *A. muricate* aqueous and ethanolic leaf extracts were orally administered for 5 days orally at doses of 187.5, 375, and 750 mg/kg and no deaths were recorded during the study. Some studies have reported the toxicity of *A. muricata*. De Sousa et al. evaluated in vivo the acute toxicity of ethanolic *A. muricata* in mice and reported a LC_50_ of 598.8 mg/kg [29]. Acésio et al. determined that the exposition of *A. muricata* doses of 1000 mg/kg or higher resulted in the death of mice 24 hr after treatment [26]. This toxicity was attributed to acetogenins. Acetogenins are the most predominant bioactive compounds of the Annonaceace family and *A. muricata* [28]. Acetogenins exert a wide range of biochemical and pharmacological properties and their most investigated effects include anti-inflammatory and cancer preventive activities [30]. The cellular growth-inhibiting activity of *A. muricata* extract is associated with the disruption of mitochondrial membrane potential, ROS generation, and G0/G1cell arrest [31].

Furthermore, the sensitivity of the in vivo MN assay in rodent peripheral blood was determined by the response of the PC group who received CP, which showed significant genotoxic effects, including short- and long-term and cytotoxic effects compared with the NC group. CP is considered a micronucleogenic agent and is widely used as a reference mutagen which has been classified as carcinogenic for animals and humans [10, 15, 17, 24, 25]. CP is a clastogenic and alkylating agent widely used in cancer chemotherapy and expresses its genotoxicity when metabolically activated by the hepatic P450 cytochrome [32, 33]. Its metabolites such as acrolein and phosphoramide mustard can interact with DNA and induce the formation of DNA adducts that cause oxidative DNA damage [32, 33]. The normal antioxidant system can be destroyed by active metabolites of CP, resulting in the accumulation of reactive oxygen species that can cause DNA strand breaks and increase the generation of promutagenic DNA adducts [32, 33].

In the antigenotoxicity evaluation of this study, the combination of *A. muricata* extract with CP, the aqueous and ethanolic extract at the three different doses tested, reduced the DNA damage induced by the mutagen decreasing the MNPCE and MNE frequencies of up to 60% in mouse peripheral blood.

The antigenotoxic effect of *A. muricata* leaf extracts observed in this study can be explained by the presence of different phytochemical compounds of the plant [5, 7, 26]. Gavamukulya et al., through phytochemical screening on leaf extracts of *A. muricata*, determined the presence of various secondary metabolites, including flavonoids and phenols, and they were present in both the aqueous and ethanolic leaf extracts [5]. Phenolic compounds are well known for their antioxidant, antimutagenic, and antitumor activities [5], and some studies have underlined a specific class such as flavonoids [34]. Also, these compounds are well known for their antimutagenic and antitumor activities [34]. On the other hand, some studies demonstrated flavonoids' anticlastogenic and antimutagenic activities against mutagens [35]. We suggest that the capacity to significantly decrease DNA damage of *A. muricata* leaf extracts observed in the present study may be attributed to the antioxidant activity of phenolics compounds, which, by diminishing the formation of free radicals, inhibit oxidative genetic damage [34, 35]. This observation is supported by other studies which indicate that flavonoids inhibit the production of DNA adducts by mutagens [36]. This could further explain since CP is known as an alkylating and cross-linking agent with DNA, so the antioxidant and free radical scavenging effects of flavonoids react with alkyl radical or block for cross-linking between CP and DNA. Also, it has been reported that flavonoids have inhibitory effects on the activity of cytochrome P450-meadiated metabolism of xenobiotics [37]. In the present study, these and other mechanisms may be responsible for the decreased MN frequency by flavonoid compounds induced by CP.

In the pregnant rats, the three different doses of aqueous or ethanolic leaf extract of *A. muricata* had a similar effect on MNPCE frequencies as it was observed in mice. Also, as in mice, the PCE values did not show significant differences at any of the sampling times. Considering that in pregnant rats, the doses of *A. muricata* leaf extracts were administered orally during pregnancy, the differences between rat weights during pregnancy, the number of offspring per litter, and pup weight at birth did not differ between groups.

In the literature, there is no information about the potential genotoxic or cytotoxic effects of *A. muricata* leaf extracts during the gestational period. We evaluated for the first time the genotoxic effect of *A. muricata* aqueous and ethanolic leaf extracts administered orally to pregnant rats directly and assessed the effect on their newborn pups, since the neonate rat is a very sensitive model to detect genotoxicity by the transplacental MN assay [18]. This assay can evaluate whether the compound administrated to the mother could cause harmful effects on the fetus due to an increase in the MN frequency in the peripheral blood of neonates. This increase provides information about the test agent's possible genotoxicity and teratogenic potential [13, 18, 38].

The results of the genotoxic and cytotoxic effect in newborn rats after transplacental exposure to *A. muricata* aqueous and ethanolic leaf extracts were like those observed in the adult mouse peripheral blood, i.e., transplacental exposure to *A. muricata* leaf extracts did not increase MNE or MNPCE frequencies or decrease the proportion of PCEs in peripheral blood of newborn rats. The number of PCEs did not show differences between experimental and negative control groups under test conditions. This finding may be due to the doses used in the oral exposure experiment in pregnant rats to evaluate possible genotoxic and cytotoxic effects in adult rats and in the litters exposed during the gestational period.

The genotoxic evaluation of medicinal plants, employing an in vivo transplacental model, might be considered since herbal remedies contain phytochemical compounds that can be toxic to pregnant women and their fetuses since some plant components can cross the placental barrier [39]; for instance, safflower (*Carthamus tinctorius*) causes eyelid defects or brain, renal, and hepatic toxicity [40]; hydroalcoholic extract of *Stachys lavandulifolia* (lamb's ears) may cause a significant decrease in height and weight as well as hepatotoxicity [41, 42]. Moreover, the literature cites that synthetic flavonoids cross the placenta in pregnant rats and accumulate in the fetal compartment, including the fetal brain [43].

## 5. Conclusions

In conclusion, it is necessary to summarize the results of our experiment, which was demonstrated under the experimental conditions used in this study that *A. muricata* aqueous and ethanolic leaf extracts (187.5, 375, and 750 mg/kg) administered orally for 5 days did not present genotoxic effects in two adult rodent models. Regarding genotoxicity induced by CP, both leave extracts of *A. muricata* rendered significant protection against DNA damage, and this protective effect could be attributed to the antioxidant activity of *A. muricata*. Furthermore, no evidence of genotoxic effects was found when the compound was administered orally to pregnant rats during the final gestation phase, as assessed both in the mother, exposed directly, and in neonatal neonates exposed transplacentally. The importance of these findings is to contribute to the favorable safety profile of the leaf extract of *A. muricata* and further investigate this plant's beneficial pharmacological properties and involving mechanisms. Also, this plant extract possesses an antigenotoxic effect and may be a potential source of safe, effective, and cheap antioxidant drugs.

## Figures and Tables

**Table 1:** Frequencies of MNPCEs/1,000 PCEs in mouse peripheral blood study groups treated with different doses of *A. muricata* aqueous and ethanolic leaf extracts at the different sampling times.

MNPCEs/1000 PCEs							
Sampling time							
	Treatments	0 h	24 h	48 h	72 h	96 h	120 h
NC *n*: 5	Sterile water	3.00 ± 0.89	3.20 ± 1.24	2.00 ± 0.44	3.00 ± 0.83	3.00 ± 0.63	2.60 ± 0.24
			NS	NS	NS	NS	NS

Aqueous extract *A. muricata* *n*: 5	187.5 mg/kg	3.20 ± 0.66	1.60 ± 0.67	3.80 ± 1.39	4.00 ± 0.70	2.80 ± 0.37	2.00 ± 0.44
			NS	NS	NS	NS	NS
	375 mg/kg	4.00 ± 0.44	2.40 ± 0.51	4.60 ± 0.68	4.00 ± 0.44	1.60 ± 0.92	5.90 ± 1.93
			NS	NS	NS	NS	NS
	750 mg/kg	1.60 ± 0.51	3.20 ± 0.58	3.60 ± 1.07	5.00 ± 0.54	3.20 ± 0.49	2.80 ± 0.58
			NS	NS	NS	NS	NS

EtOH Extract *A. muricata* *n*: 5	187.5 mg/kg	3.60 ± 0.67	2.20 ± 0.58	3.60 ± 1.06	4.20 ± 0.58	2.80 ± 0.58	4.00 ± 0.54
			NS	NS	NS	NS	NS
	375 mg/kg	2.80 ± 0.97	2.60 ± 0.60	3.20 ± 0.37	4.00 ± 0.70	3.60 ± 0.81	3.00 ± 0.94
			NS	NS	NS	NS	NS
	750 mg/kg	2.60 ± 0.78	2.60 ± 0.51	2.60 ± 1.12	3.40 ± 0.74	3.40 ± 0.40	2.40 ± .60
			NS	NS	NS	NS	NS

PC *n*: 5	CF	3.40 ± 0.67	9.00 ± 1.04	11.60 ± 1.83	19.00 ± 3.03	11.60 ± 2.96	6.20 ± 1.20
	60 mg/kg		0.001^∗^	0.017^∗^	0.009^∗^	0.001^∗^	0.009^∗^

Data are expressed as mean ± standard deviation per group. Intragroup comparisons were performed between baseline samples (0 h) against the following sampling times: 24, 48, 72, 96, and 120 h (ANOVA, Bonferroni post hoc test, ^∗^*p* < 0.05). *A. muricata*: *Annona muricata*; CP: cyclophosphamide; MNPCEs: micronucleated polychromatic erythrocytes; *n*: mice per group; NC: negative control; NS: not significant; PC: positive control; PCEs: polychromatic erythrocytes.

**Table 2:** Frequencies of MNEs/10000 TEs in mouse peripheral blood study groups treated with different doses of *A. muricata* aqueous and ethanolic leaf extracts at the different sampling times.

MNEs/10000 TEs							
Sampling time							
	Treatments	0 h	24 h	48 h	72 h	96 h	120 h
NC *n*: 5	Sterile water	20.80 ± 2.05	24.6 ± 0.98	20.40 ± 1.20	19.20 ± 1.68	22.60 ± 1.32	22.20 ± 1.49
			NS	NS	NS	NS	NS

Aqueous extract *A. muricata* *n*: 5	187.5 mg/kg	22.80 ± 4.58	23.40 ± 1.74	25.00 ± 2.47	21.60 ± 2.15	22.00 ± 3.11	22.60 ± 2.11
			NS	NS	NS	NS	NS
	375 mg/kg	22.20 ± 2.59	23.80 ± 1.98	20.80 ± 1.49	22.20 ± 1.80	23.40 ± 0.67	20.60 ± 1.50
			NS	NS	NS	NS	NS
	750 mg/kg	21.00 ± 3.57	22.40 ± 2.13	24.00 ± 3.55	29.00 ± 3.27	29.20 ± 3.26	17.40 ± 2.65
			NS	NS	NS	NS	NS

EtOH extract *A. muricata* *n*: 5	187.5 mg/kg	20.20 ± 1.46	30.40 ± 5.44	26.20 ± 3.12	23.40 ± 2.40	21.80 ± 2.72	21.00 ± 1.04
			NS	NS	NS	NS	NS
	375 mg/kg	22.20 ± 2.53	22.40 ± 2.80	21.60 ± 1.91	20.20 ± 2.49	22.20 ± 2.01	17.20 ± 1.49
			NS	NS	NS	NS	NS
	750 mg/kg	15.00 ± 2.32	23.80 ± 2.08	22.20 ± 1.88	23.00 ± 1.94	22.60 ± 1.03	21.00 ± 1.04
			NS	NS	NS	NS	NS

PC *n*: 5	CP	21.80 ± 3.16	35.20 ± 4.03	56.20 ± 3.83	60.60 ± 2.89	59.20 ± 4.46	36.00 ± 3.08
	60 mg/kg		NS	0.002^∗^	0.013^∗^	0.009^∗^	NS

Data are expressed as mean ± standard deviation per group. Intragroup comparisons were performed between baseline samples (0 h) against the following sampling times: 24, 48, 72, 96, and 120 h (ANOVA, Bonferroni post hoc test, ^∗^*p* < 0.05). *A. muricata*: *Annona muricata*; CP: cyclophosphamide; MNEs: micronucleated erythrocytes; *n*: mice per group; NC: negative control; NS: not significant; PC: positive control; TEs: total erythrocytes.

**Table 3:** Frequencies of PCEs/1000 TEs in mouse peripheral blood study groups treated with different doses of *A. muricata* aqueous and ethanolic leaf extracts at the different sampling times.

PCEs/1000 TEs							
Sampling time							
	Treatments	0 h	24 h	48 h	72 h	96 h	120 h
NC *n*: 5	Sterile water	42.60 ± 9.10	57.20 ± 5.25	60.20 ± 7.78	53.60 ± 12.40	58.40 ± 14.70	46.20 ± 5.76
			NS	NS	NS	NS	NS

Aqueous extract *A. muricata* *n*: 5	187.5 mg/kg	54.00 ± 7.32	45.00 ± 7.42	50.40 ± 6.95	39.80 ± 6.12	31.80 ± 4.60	50.80 ± 6.94
			NS	NS	NS	NS	NS
	375 mg/kg	64.80 ± 6.68	45.60 ± 7.58	65.20 ± 2.87	61.20 ± 4.69	36.80 ± 4.42	39.80 ± 4.03
			NS	NS	NS	NS	NS
	750 mg/kg	72.20 ± 19.69	55.00 ± 15.10	57.40 ± 11.12	63.00 ± 14.79	75.60 ± 15.12	44.80 ± 7.94
			NS	NS	NS	NS	NS

EtOH extract *A. muricata* *n*: 5	187.5 mg/kg	77.60 ± 10.07	53.80 ± 5.99	60.80 ± 9.50	58.60 ± 4.88	32.60 ± 4.23	55.00 ± 4.05
			NS	NS	NS	NS	NS
	375 mg/kg	65.20 ± 12.13	56.00 ± 9.91	53.40 ± 4.60	50.00 ± 2.98	46.80 ± 11.25	73.20 ± 7.87
			NS	NS	NS	NS	NS
	750 mg/kg	56.80 ± 7.48	70.60 ± 13.77	43.60 ± 10.02	52.80 ± 8.26	53.40 ± 4.22	58.00 ± 10.76
			NS	NS	NS	NS	NS

PC *n*: 5	CP	89.40 ± 10.70	50.60 ± 10.80	47.00 ± 10.65	26.60 ± 4.45	31.40 ± 5.15	36.00 ± 3.86
	60 mg/kg		NS	NS	0.002^∗^	0.032^∗^	0.033^∗^

Data are expressed as mean ± standard deviation per group. Intragroup comparisons were performed between baseline samples (0 h) against the following sampling times: 24, 48, 72, 96, and 120 h (ANOVA, Bonferroni post hoc test, ^∗^*p* < 0.05). *A. muricata*: *Annona muricata*; CP: cyclophosphamide; PCEs: polychromatic erythrocytes; n: mice per group; NC: negative control; NS: not significant; PC: positive control; TEs: total erythrocytes.

**Table 4:** Evaluation of the antigenotoxic effect and %DR of *A. muricata* aqueous and ethanolic leaf extracts against CP-induced damage in MNPCEs cell count in mouse peripheral blood.

MNPCEs/1000 PCEs							
Sampling time							
	Treatments	0 h	24 h	48 h	72 h	96 h	120 h
NC *n*: 5	Sterile water	3.00 ± 0.89	3.20 ± 1.24	1.80 ± 0.49	3.00 ± 0.84	3.00 ± 0.63	2.60 ± 0.24
			NS	NS	NS	NS	NS

PC *n*: 5	CP	3.40 ± 0.68	9.00 ± 1.05	11.60 ± 1.83	19.00 ± 3.03	11.60 ± 2.96	6.20 ± 1.20
	60 mg/kg		NS	NS	NS	NS	0.043

CP+aqueous extract *A. muricata* *n*: 5	60 mg/kg+187.5 mg/kg	4.00 ± 0.89	7.00 ± 0.63	5.60 ± 0.68	8.20 ± 0.20	7.40 ± 0.24	5.60 ± 0.68
			NS	^∗^0.001	^∗^0.001	NS	NS
	%DR		34.48%	61.22%	67.50%	48.84%	16.67%
	60 mg/kg+375 mg/kg	3.60 ± 0.68	6.20 ± 0.80	7.40 ± 0.81	9.00 ± 1.30	6.80 ± 0.86	4.00 ± 0.55
			NS	0.031^∗^	0.001^∗^	0.037^∗^	NS
	%DR		48.28%	42.86%	62.50%	55.81%	61.11%
	60 mg/kg+750 mg/kg	3.80 ± 0.49	6.80 ± 1.77	8.80 ± 0.86	10.80 ± 1.11	6.80 ± 1.11	5.80 ± 0.58
			NS	NS	NS	0.002^∗^	0.037^∗^
	%DR		37.93%	28.57%	51.25%	55.81%	11.11%

CP+EtOH extract *A. muricata* *n*: 5	60 mg/kg+187.5 mg/kg	3.40 ± 0.51	7.00 ± 0.84	5.00 ± 0.45	8.00 ± 1.14	5.40 ± 0.51	4.00 ± 0.32
			NS	NS	0.001^∗^	0.001^∗^	0.004^∗^
	%DR		34.48%	68.75%	68.75%	72.09%	61.11%
	60 mg/kg+375 mg/kg	2.60 ± 0.24	6.60 ± 1.03	7.00 ± 1.38	8.20 ± 1.24	6.00 ± 0.84	3.60 ± 0.98
		NS	NS	0.015^∗^	0.001^∗^	0.011^∗^	NS
	%DR		41.38%	46.94%	67.50%	65.12%	72.22%
	60 mg/kg+750 mg/kg	2.80 ± 0.58	7.60 ± 1.36	6.80 ± 0.66	7.80 ± 0.66	5.80 ± 0.58	5.00 ± 0.45
			NS	NS	0.011^∗^	0.001^∗^	0.008^∗^
	%DR		24.14%	48.98%	70.00%	67.44%	33.33%

Data are expressed as mean ± standard deviation per group. One-way ANOVA, followed by the Dunnett *t*-test for multiple post hoc pairwise comparisons versus the PC, was employed to correct the significance (^∗^*p* < 0.05) values for intergroup analysis. %DR: percentage of damage reduction; CP: cyclophosphamide; MNPCEs: micronucleated polychromatic erythrocytes; n: mice per group; NC: negative control; NS: not significant; PC: positive control; PCEs: polychromatic erythrocytes.

**Table 5:** Evaluation of the antigenotoxic effect and %DR of *A. muricata* aqueous and ethanolic leaf extracts against CP-induced damage in MNEs cell count in mouse peripheral blood.

MNEs/10,000 TEs							
Sampling time							
	Doses	0 h	24 h	48 h	72 h	96 h	120 h
NC *n*: 5	Sterile water	20.80 ± 2.06	24.60 ± 0.98	20.40 ± 1.21	19.20 ± 1.69	22.60 ± 1.33	22.20 ± 1.50

PC *n*: 5	CP	31.80 ± 3.17	35.20 ± 4.03	36.20 ± 3.84	58.60 ± 2.89	49.20 ± 6.47	36.00 ± 3.08

CP+aqueous extract *A. muricata* *n*: 5	60 mg/kg+187.5 mg/kg	26.80 ± 2.82	28.60 ± 4.18	26.00 ± 0.95	37.00 ± 4.62	26.80 ± 2.78	29.60 ± 4.37
			NS	NS	0.010^∗^	0.001^∗^	0.001^∗^
	%DR		62.26%	64.56%	54.82%	84.21%	46.38%
	60 mg/kg+375 mg/kg	26.00 ± 4.18	29.60 ± 2.32	23.40 ± 1.17	24.40 ± 2.01	27.40 ± 3.26	27.40 ± 1.03
			NS	NS	0.001^∗^	0.001^∗^	0.001^∗^
	%DR		52.83%	81.01%	86.80%	81.95%	62.32%
	60 mg/kg+750 mg/kg	25.60 ± 1.50	30.20 ± 1.93	26.80 ± 1.28	31.80 ± 1.28	30.80 ± 69.17	30.40 ± 3.28
			NS	NS	0.010^∗^	0.001^∗^	0.002^∗^
	%DR		47.17%	59.49%	68.02%	69.17%	40.58%

CP+EtOH extract *A. muricata* *n*: 5	60 mg/kg+187.5 mg/kg	24.40 ± 2.20	31.40 ± 0.98	29.20 ± 1.24	36.60 ± 3.91	31.80 ± 0.86	27.60 ± 0.68
			NS	NS	NS	0.001^∗^	0.003^∗^
	%DR		35.85%	44.30%	55.84%	65.41%	60.87%
	60 mg/kg+375 mg/kg	25.60 ± 2.40	28.20 ± 1.28	25.00 ± 2.19	32.80 ± 1.83	33.80 ± 1.36	27.40 ± 1.44
		NS	NS	0.002^∗^	0.001^∗^	0.009^∗^	NS
	%DR		66.04%	70.89%	65.48%	57.89%	62.32%
	60 mg/kg+750 mg/kg	21.00 ± 1.79	29.00 ± 1.95	28.80 ± 1.96	36.00 ± 1.92	37.60 ± 2.84	31.60 ± 2.99
			0.037^∗^	NS	0.059^∗^	0.001^∗^	NS
	%DR		58.49%	46.84%	57.36%	43.61%	31.88%

Data are expressed as mean ± standard deviation per group. One-way ANOVA followed by the Dunnett *t*-test for multiple post hoc pairwise comparisons versus the PC was employed to correct the significance (^∗^*p* < 0.05) values for intergroup analysis. %DR: percentage of damage reduction; CP: cyclophosphamide; MNEs: micronucleated erythrocytes; *n*: mice per group; NC: negative control; NS: not significant; PC: positive control; TEs: total erythrocytes.

**Table 6:** Frequencies of MNPCEs/1,000 PCEs in pregnant rats peripheral blood study groups treated with different doses of *A. muricata* aqueous and ethanolic leaf extracts at the different sampling times.

MNPCEs/1000 PCEs							
Sampling time							
	Treatments	0 h	24 h	48 h	72 h	96 h	120 h
NC *n*: 5	Sterile water	2.00 ± 0.44	1.40 ± 0.51	2.20 ± .20	2.00 ± 0.31	2.40 ± 0.67	3.00 ± 0.83
			NS	NS	NS	NS	NS

Aqueous extract *A. muricata* *n*: 5	187.5 mg/kg	1.20 ± 0.58	1.60 ± 0.81	3.20 ± 0.49	2.00 ± 0.63	1.80 ± 0.49	1.80 ± 0.80
			NS	NS	NS	NS	NS
	375 mg/kg	2.60 ± 0.51	1.40 ± 0.40	4.60 ± 0.24	3.40 ± .92	3.20 ± 1.20	2.80 ± 0.66
			NS	NS	NS	NS	NS
	750 mg/kg	2.40 ± 0.40	1.40 ± 0.51	2.80 ± 1.11	2 ± 0.89	1.80 ± 0.37	1.60 ± 0.40
			NS	NS	NS	NS	NS

EtOH extract *A. muricata* *n*: 5	187.5 mg/kg	1.60 ± 0.24	2.00 ± 0.54	2.40 ± 0.51	2.20 ± 0.37	2.20 ± 0.37	1.80 ± 0.49
			NS	NS	NS	NS	NS
	375 mg/kg	1.00 ± 0.44	1.60 ± 0.51	3.60 ± 0.81	1.80 ± 0.58	1.40 ± 0.24	0.40 ± 0.24
			NS	NS	NS	NS	NS
	750 mg/kg	1.00 ± 0.31	1.40 ± 0.24	1.60 ± 0.51	2.80 ± 0.37	2.60 ± 0.81	3.00 ± 0.77
			NS	NS	NS	NS	NS

PC *n*: 5	CP	2.56 ± 0.29	5.50 ± 1.73	6.75 ± 1.70	6.00 ± 2.58	10.75 ± 2.21	18.50 ± 4.35
	60 mg/kg		NS	NS	NS	0.001^∗^	0.001^∗^

Data are expressed as mean ± standard deviation per group. Intragroup comparisons were performed between baseline samples (0 h) against the following sampling times: 24, 48, 72, 96, and 120 h (ANOVA Bonferroni test post hoc for multiple comparisons, ^∗^*p* < 0.05). *A. muricata: Annona muricata*; CP: cyclophosphamide; MNPCEs: micronucleated polychromatic erythrocytes; *n*: mice per group; NC: negative control; NS: not significant; PCEs: polychromatic erythrocytes; PC: positive control.

**Table 7:** Frequencies of PCEs/1000 TEs in pregnant rat peripheral blood study groups treated with different doses of A. muricata aqueous and ethanolic leaf extracts at the different sampling times.

PCEs/1000 TEs							
Sampling time							
	Treatment	0 hrs	24 hrs	48 hrs	72 hrs	96 hrs	120 hrs
NC *n*: 5	Sterile water	45.40 ± 5.24	45.20 ± 6.06	34.80 ± 2.26	47.00 ± 2.93	42.00 ± 3.64	47.20 ± 7.01
			NS	NS	NS	NS	NS

Aqueous extract *A. muricata* *n*: 5	187.5 mg/kg	45.40 ± 3.62	49.60 ± 5.14	46.80 ± 6.87	46.00 ± 5.97	46.00 ± 3.70	49.40 ± 7.59
			NS	NS	NS	NS	NS
	375 mg/kg	62.40 ± 6.44	53.20 ± 8.80	50.20 ± 7.78	58.00 ± 7.23	55.20 ± 7.29	40.00 ± 6.53
			NS	NS	NS	NS	NS
	750 mg/kg	58.80 ± 9.57	54.60 ± 4.70	53.60 ± 6.65	40.80 ± 3.77	36.40 ± 4.47	31.60 ± 2.71
			NS	NS	NS	NS	NS

EtOH extract *A. muricata* *n*: 5	187.5 mg/kg	55.20 ± 6.09	40.40 ± 4.15	45.80 ± 5.97	44.40 ± 5.05	48.20 ± 6.17	37.60 ± 2.69
			NS	NS	NS	NS	NS
	375 mg/kg	47.40 ± 4.09	47.60 ± 3.98	58.60 ± 6.58	51.60 ± 2.24	46.00 ± 4.50	44.20 ± 3.85
			NS	NS	NS	NS	NS
	750 mg/kg	45.00 ± 1.44	40.80 ± 3.68	35.00 ± 6.05	41.20 ± 3.87	37.40 ± 1.77	35.80 ± 1.59
			NS	NS	NS	NS	NS

PC *n*: 5	CP	38.17 ± 8.86	38.17 ± 3.12	40.00 ± 11.67	34.50 ± 9.39	18.33 ± 5.68	5.83 ± 1.47
	60 mg/kg		NS	NS	NS	0.007^∗^	0.005^∗^

Data are expressed as mean ± standard deviation per group. Intragroup comparisons were performed between baseline samples (0 h) against the following sampling times: 24, 48, 72, 96, and 120 h (ANOVA Bonferroni test post hoc for multiple comparisons, ^∗^*p* < 0.05). *A. muricata: Annona muricata*; CP: cyclophosphamide; PCEs: Polychromatic erythrocytes; *n*: mice per group; NC: negative control; NS: not significant; PC: positive control; TEs: total erythrocytes.

**Table 8:** Frequencies of MNPCEs, MNEs, and PCEs from neonate peripheral blood of the pregnant rats study groups.

Groups	Treatments	*n*	MNPCEs	MNEs	PCEs
NC	Sterile water	6	5.40 ± 0.39	12.30 ± 0.43	88.83 ± 3.51
			NS	NS	NS

Aqueous extract *A. muricata*	187.5 mg/kg	6	5.93 ± 0.45	12.40 ± 0.88	84 ± 3.51
			NS	NS	NS
	375 mg/kg	6	5.57 ± 0.44	12.90 ± 0.81	105.10 ± 4.98
			NS	NS	NS
	750 mg/kg	6	5.53 ± 0.35	10.03 ± 0.79	102.23 ± 3.23
			NS	NS	NS

EtOH extract *A. muricata*	187.5 mg/kg	6	7.87 ± 0.66	16.50 ± 1.16	106.03 ± 5.10
			NS	NS	NS
	375 mg/kg	6	6.43 ± 0.38	12.03 ± 0.89	91.17 ± 2.81
			NS	NS	NS
	750 mg/kg	6	7.77 ± 0.54	14.47 ± 1.95	102.23 ± 0.89
			NS	NS	NS

PC	CP	6	27.60 ± 1.94	112.70 ± 7.69	61.73 ± 3.23
	60 mg/kg		0.0001^∗^	0.0001^∗^	0.0001^∗^

Data are expressed as mean ± standard deviation per group. *A. muricata* aqueous and EtOH extracts were administered orally to pregnant rats. One-way ANOVA followed by the Dunnett *t*-test for multiple post hoc pairwise comparisons versus the NC was employed to correct the significance values for intergroup analysis (^∗^*p* < 0.05). CP: cyclophosphamide; MNEs: micronucleated erythrocytes/1000 TEs; TE: total erythrocytes; MNPCEs: micronucleated polychromatic erythrocytes/1000 PCEs; *n*: sample size (pregnant rats/6 neonates per dam); NC: negative control; NS: not significant; PC: positive control; PCEs: polychromatic erythrocytes/1000 TEs; TEs: total erythrocytes.

## Data Availability

The raw data required to reproduce these findings cannot be shared at this time as the data is also forms part of an ongoing study.

## Supplementary Materials

Supplementary Materials The results of the phytochemical screening by a colorimetric test of the aqueous and ethanolic leaf extract of *A. muricata* are table. The phytochemical screening showed that contained flavonoids, among which are the flavones, chalcones, xanthones, quinones, cardiac glycosides, and coumarins.
