# Supplementary material for: Evaluation of Genotoxic Effect and Antigenotoxic Potential against DNA Damage of the Aqueous and Ethanolic Leaf Extracts of Annona muricata Using an In Vivo Erythrocyte Rodent Micronucleus Assay
Source: Biomed Res Int. 2022 Dec 16;2022:9554011. doi: 10.1155/2022/9554011 (PMC9788883; doi:10.1155/2022/9554011)
Supplement: Supplementary Materials — The results of the phytochemical screening by a colorimetric test of the aqueous and ethanolic leaf extract of A. muricata are table. The phytochemical screening showed that contained flavonoids, among which are the flavones, chalcones, xanthones, quinones, cardiac glycosides, and coumarins. [file 9554011.f1.docx]

**Supplementary material**

The results of the phytochemical screening by a colorimetric test of the aqueous and ethanolic leaves extract of *A. muricata* are in table . The phytochemical screening showed that contained flavonoids, among which are the flavones, chalcones, xanthones, quinones, cardiac glycosides and coumarins.

**Table . Phytochemical screening of the aqueous and ethanolic leaves extract of *A. muricata***

| **Compound** | **Aqueous extract** | **Ethanolic extract** |
| --- | --- | --- |
| Alkaloids | - | - |
| Flavonoids | - | - |
| Flavones | + | - |
| Chalcones | - | + |
| Xanthones | - | + |
| Cardiac Glycosides | + | + |
| Quinones | + | - |
| saponins | + | - |
| Tannins | - | - |
| Coumarins | + | + |
| Sesquiterpene lactones | - | - |
| Benzoquinones / Anthrone | -/- | -/- |

Note: (+) = present ; (-) = absent.
